# Supplementary material for: Effects of nettle slurry (Urtica dioica L.) used as foliar fertilizer on potato (Solanum tuberosum L.) yield and plant growth
Source: PeerJ. 2018 May 7;6:e4729. doi: 10.7717/peerj.4729 (PMC5944444; doi:10.7717/peerj.4729)
Supplement: Supplemental Information 2 [file peerj-06-4729-s002.pdf]

Slurry / Parameter	pH	EC	OM (%)	OOM(%)	Ash(%)	%K_2O (p/V)	%P_2O_5 (p/V)	Total N (%)	Protein (%)
Other Urtica slurry	4.34	5.55	1.69	1.8	0.29	0.069	0,017	0,037	0,23
