# Supplementary figures and images for: Effects of nettle slurry (Urtica dioica L.) used as foliar fertilizer on potato (Solanum tuberosum L.) yield and plant growth
Source: PeerJ. 2018 May 7;6:e4729. doi: 10.7717/peerj.4729 (PMC5944444; doi:10.7717/peerj.4729)

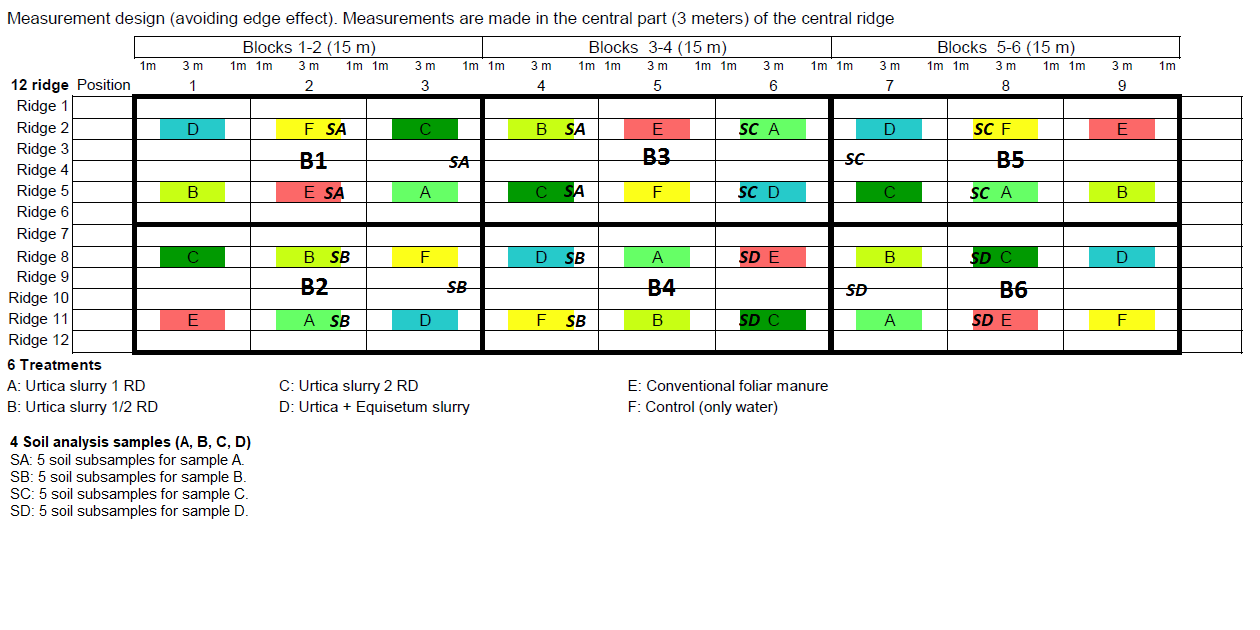

Supplement: Supplemental Information 5 [file peerj-06-4729-s005.png]

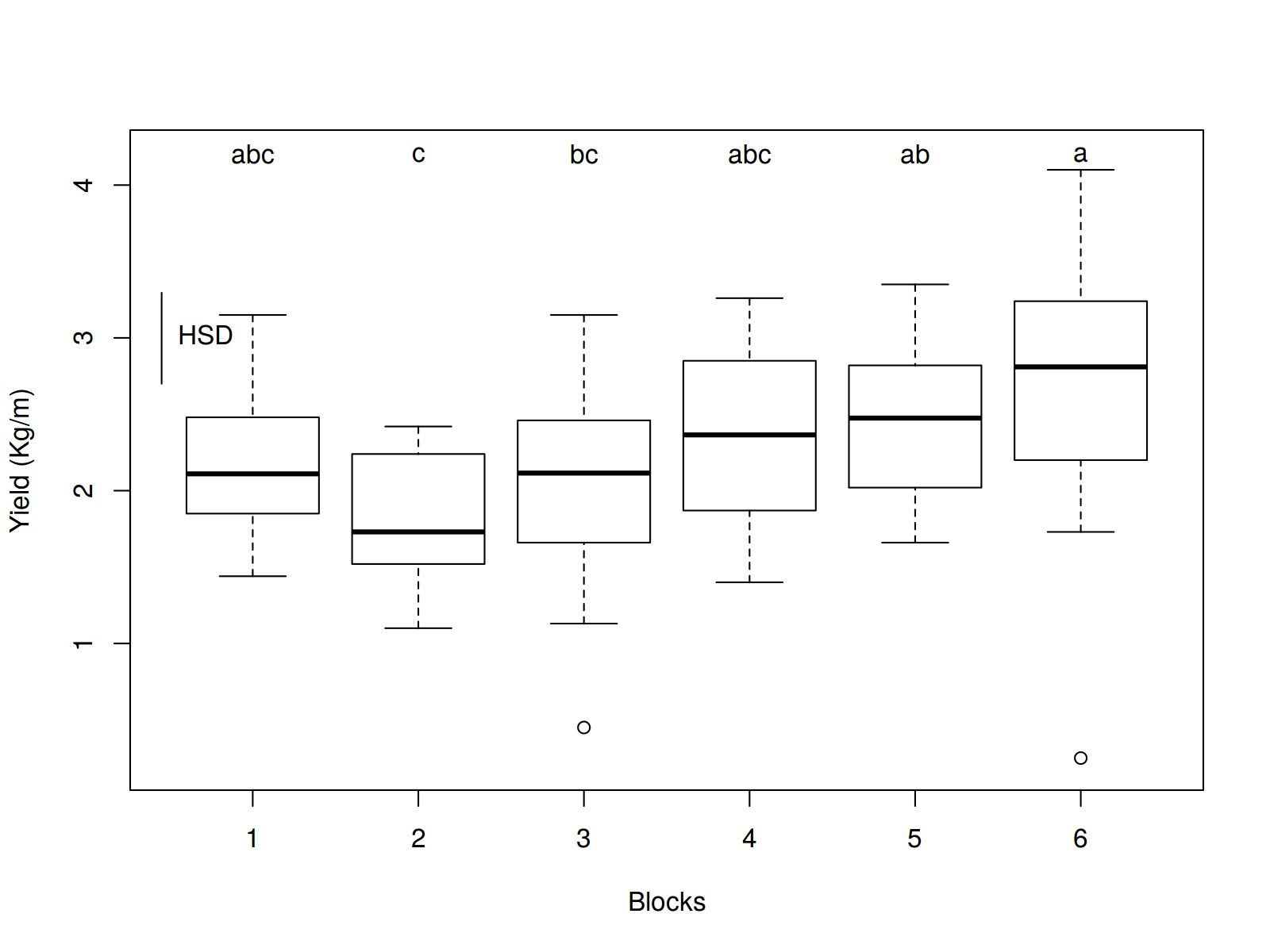

Supplement: Supplemental Information 8 [file peerj-06-4729-s008.png]

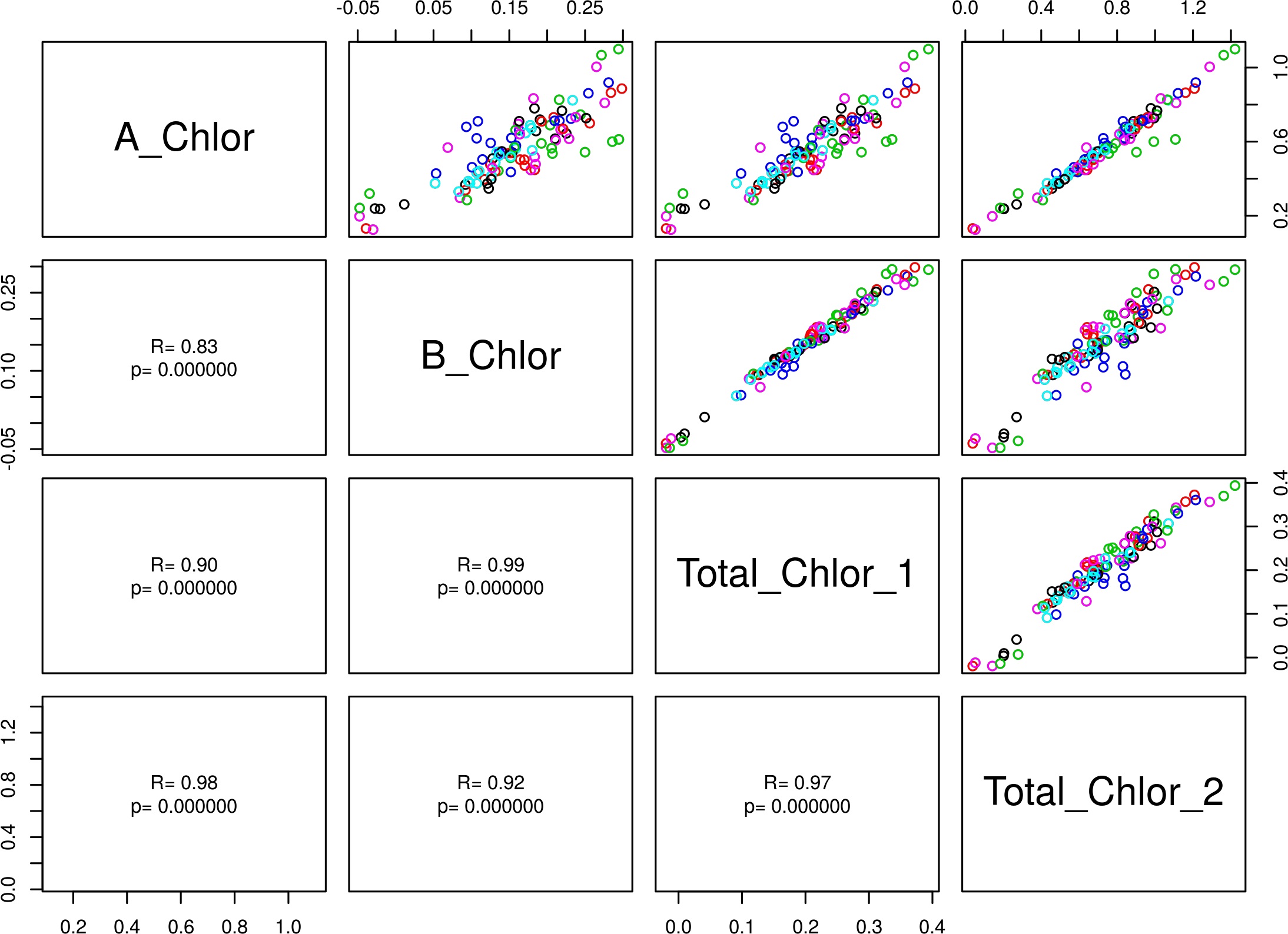

Supplement: Supplemental Information 9 [file peerj-06-4729-s009.jpg]

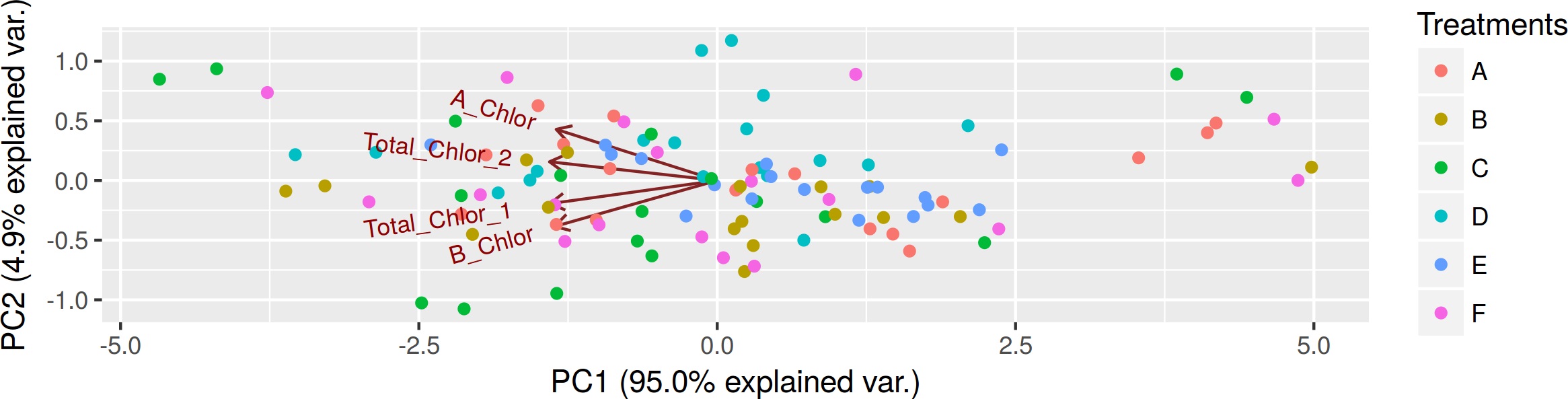

Supplement: Supplemental Information 10 [file peerj-06-4729-s010.jpg]
