# Supplementary material for: Effects of nettle slurry (Urtica dioica L.) used as foliar fertilizer on potato (Solanum tuberosum L.) yield and plant growth
Source: PeerJ. 2018 May 7;6:e4729. doi: 10.7717/peerj.4729 (PMC5944444; doi:10.7717/peerj.4729)

Effect of sampler

The analysis of variance for the effect of Sampler on yield shows NO significant differences among treatments (p = 0.76972, R^2 = 0.02429).

Table 1: Table 0: Summary of the mean yields achieved by each sampler (Kg/m) with the Analysis of variance values and post-hoc Tukey test. In Treatments RD, recommended dosage; N, number of repetitions; HSD, post-hoc Tukey test Honestly Significant Difference; Sd, standard deviation; Se, standard error; standardized Skewness & Kurtosis; DF, degrees of freedom

Tr	N	mean	HSD	sd	se	skew	kurtosis	Shapiro
YieA	36	2.4628	a	0.58261	0.09710	0.53892	-0.44914	0.15739
YieB	36	2.1369	a	0.75080	0.12513	0.32754	0.34508	0.83200
YieC	36	2.1256	a	0.62446	0.10408	-0.52254	0.96815	0.40973

	Df	Sum Sq	Mean Sq	F value	Pr(>F)	HSD	eta.sq
YSampler	2	2.6402	1.32010	3.0627	0.05096	0.367891618581786	0.05512
Residuals	105	45.2580	0.43103	NA	NA		NA

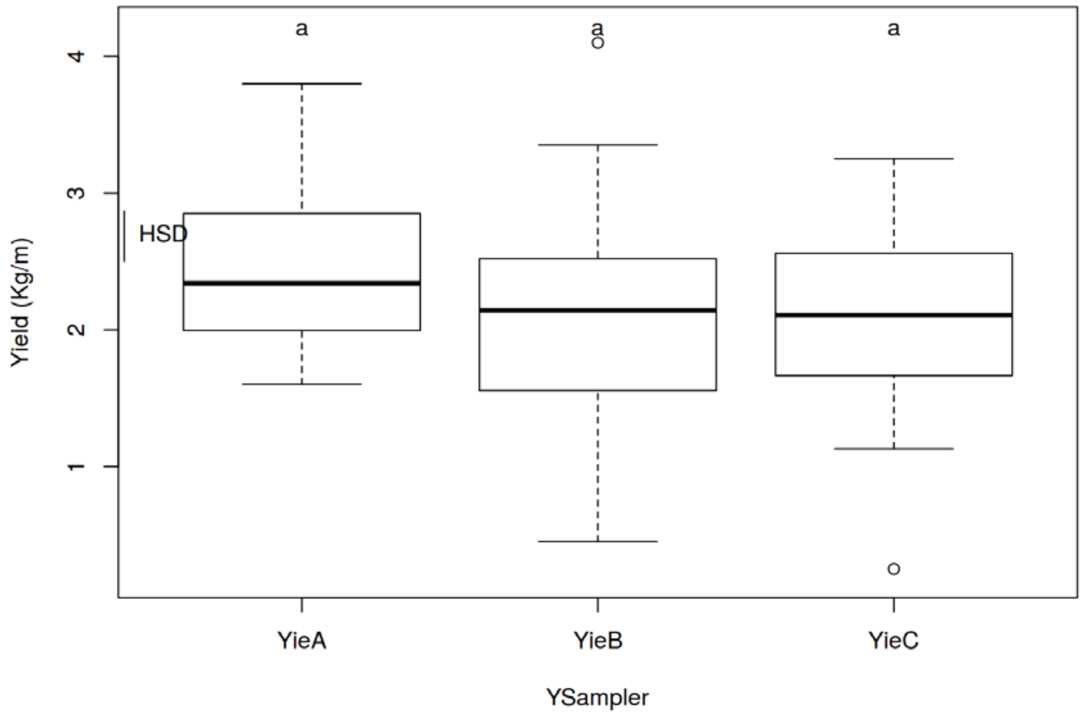

Supplement: Supplemental Information 6 [file peerj-06-4729-s006.pdf]
