# Supplementary material for: Effects of nettle slurry (Urtica dioica L.) used as foliar fertilizer on potato (Solanum tuberosum L.) yield and plant growth
Source: PeerJ. 2018 May 7;6:e4729. doi: 10.7717/peerj.4729 (PMC5944444; doi:10.7717/peerj.4729)
Supplement: Supplemental Information 7 [file peerj-06-4729-s007.pdf]

Treatment A

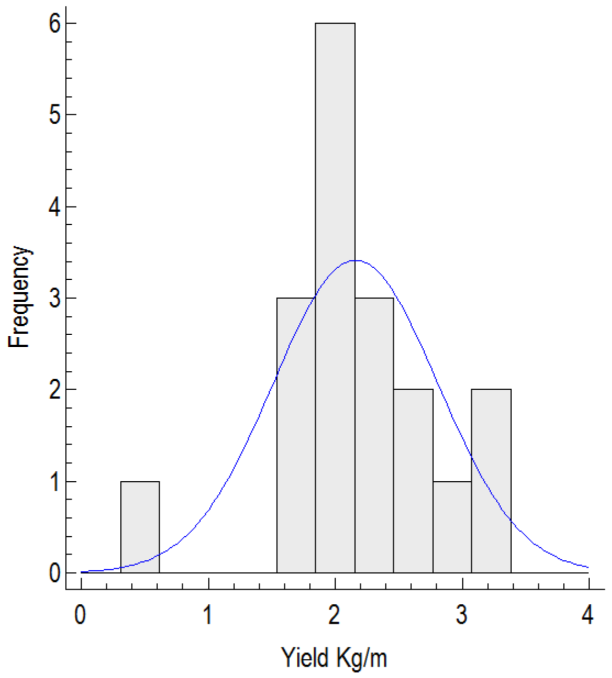

Blue line represents a normal distribution
Shapiro-Wilk p-value = 0.263

Treatment B

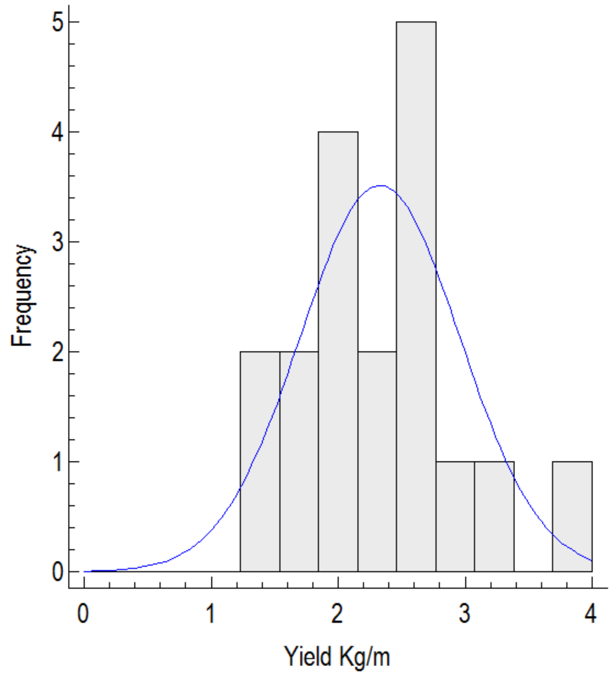

Blue line represents a normal distribution
Shapiro-Wilk p-value = 0.680

Treatment C

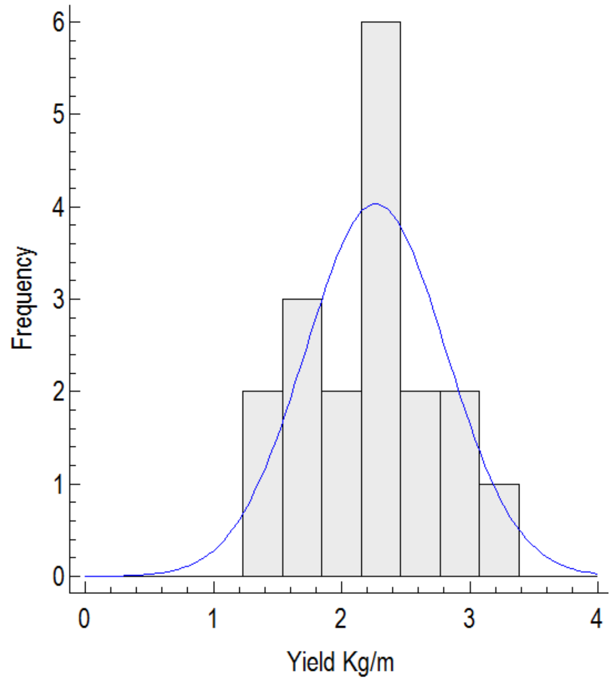

Blue line represents a normal distribution
Shapiro-Wilk p-value = 0.716

Treatment D

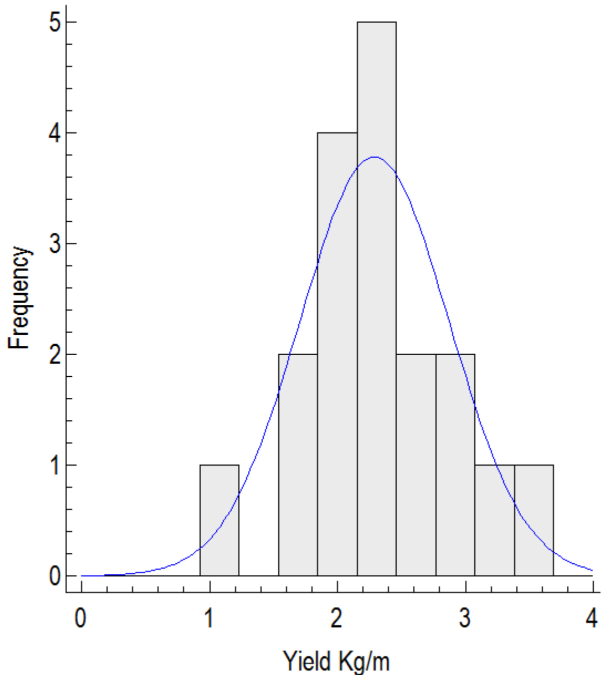

Blue line represents a normal distribution
Shapiro-Wilk p-value = 0.994

Treatment E

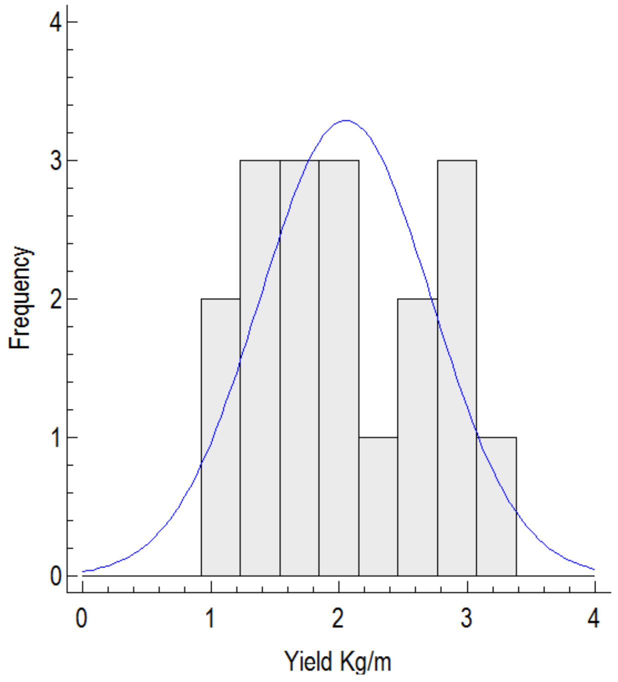

Blue line represents a normal distribution
Shapiro-Wilk p-value = 0.287

Treatment F

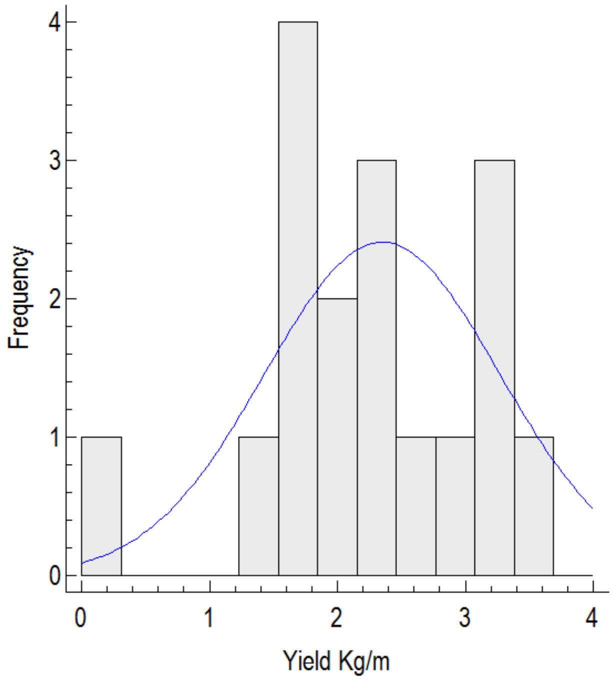

Blue line represents a normal distribution
Shapiro-Wilk p-value = 0.815
